# Assessing Finger Joint Biomechanics by Applying Equal Force to Flexor Tendons *In Vitro* Using a Novel Simultaneous Approach

**DOI:** 10.1371/journal.pone.0160301

**Published:** 2016-08-11

**Authors:** Tai-Hua Yang, Szu-Ching Lu, Wei-Jr Lin, Kristin Zhao, Chunfeng Zhao, Kai-Nan An, I-Ming Jou, Pei-Yuan Lee, Li-Chieh Kuo, Fong-Chin Su

**Affiliations:** 1 Department of Biomedical Engineering, National Cheng Kung University, Tainan, Taiwan; 2 Department of Occupational Therapy, National Cheng Kung University, Tainan, Taiwan; 3 Department of Orthopedic, National Cheng Kung University, Tainan, Taiwan; 4 Medical Device Innovation Center, National Cheng Kung University, Tainan, Taiwan; 5 Rehabilitation Medicine Research Center, Department of Physical Medicine and Rehabilitation, Mayo Clinic, Rochester, Minnesota, United States of America; 6 Biomechanics & Tendon and Soft Tissue Biology Laboratory, Division of Orthopedic Research, Mayo Clinic, Rochester, Minnesota, United States of America; 7 Department of Orthopedics, Show Chwan Memorial Hospital, Changhua, Taiwan; University of Chicago, UNITED STATES

## Abstract

**Background:**

The flexor digitorum superficialis (FDS) and flexor digitorum profundus (FDP) are critical for finger flexion. Although research has recently focused on these tendons’ coactivity, their contributions in different tasks remain unclear. This study created a novel simultaneous approach to investigate the coactivity between the tendons and to clarify their contributions in different tasks.

**Methods:**

Ten human cadaveric hands were mounted on our custom frame with the FDS and FDP of the third finger looped through a mechanical pulley connected to a force transducer. Joint range of motion, tendon excursion and loading force were recorded during individual joint motion and free joint movement from rest to maximal flexion. Each flexor tendon’s moment arm was then calculated.

**Results:**

In individual motions, we found that the FDP contributed more than the FDS in proximal interphalangeal (PIP) joint motion, with an overall slope of 1.34 and all FDP-to-FDS excursion (P/S) ratios greater than 1.0 with force increase. However, the FDP contributed less than the FDS in metacarpophalangeal (MCP) joint motion, with an overall slope of 0.95 and P/S ratios smaller than 1.0 throughout the whole motion except between 1.9% and 13.1% force. In free joint movement, the FDP played a greater role than the FDS, with an overall ratio of 1.37 and all P/S ratios greater than 1.0.

**Conclusions:**

The new findings include differences in finger performance and excursion amounts between the FDS and FDP throughout flexion. Such findings may provide the basis for new hand models and treatments.

## Introduction

Flexor tendon injury is one of the most common hand conditions that limit hand use [1, 2]. Anatomically, the flexor digitorum profundus (FDP) inserts into the distal phalange’s base, and the flexor digitorum superficialis (FDS) inserts into the middle phalange’s base. A sheath and pulley system prevents these tendons from bowstringing during flexion. This integrated system promotes force transmission efficiency in flexion by optimizing the tendons’ gliding and excursion and keeping the moment arm between the joint axes and the tendons more constant, while the FDP flexes the metacarpophalangeal (MCP), proximal interphalangeal (PIP) and distal interphalangeal (DIP) joints and the FDS flexes the MCP and PIP joints [3, 4]. This system may function poorly after hand injuries, especially because adhesion may occur during healing. Different exercises have been developed to prevent this problem. In 1987, Wehbé demonstrated that a series of exercises that put the fingers in different postures lowered the chance of adhesion between the FDS and FDP by causing them to move separately and reach maximal excursion individually, something the traditional exercises failed to achieve [5]. However, the precise relationship between the FDS and FDP in flexion remains unknown, and such knowledge could benefit the further development of optimal treatment.

A biomechanical model is important for understanding the function of these structures and the relationship between the tendons [5, 6]. To investigate the tendon’s mechanical efficiency, many researchers have followed Landsmeer’s equation (Eq 1), where *M* = instantaneous moment arm, *E* = tendon excursion and *θ* = joint angle [6–9].

$$M=\frac{dE}{d\theta }$$ (1)

This equation, confirmed as reliable in multiple situations for measuring both the extensor and flexor tendons, allowed us to determine the moment arm without needing difficult geometric measurements [9–11].

Early finger biomechanics studies used an individual approach, testing the kinetic and kinematic changes of the FDS and FDP individually. Some of these studies assumed the FDS is completely inactive or triggered just slightly early in flexion. Therefore, they gathered evidence only from the FDP [12–15]. Later studies, however, analyzed both the FDS and FDP and found that both tendons are active, although these studies did not explore the specific relationship between the tendons. These studies further defined these tendons’ coordination with a “simultaneous approach,” testing them together in *vivo* [16–18]. However, previous studies were all performed in *vivo* and therefore were limited to examining tendon gliding postures, which cannot clarify the specific kinetics or kinematics of the tendons’ simultaneous function [5, 12, 16–21]. Our main purpose was to use a novel simultaneous approach to explore the two tendons’ contributions by determining finger joint angle and tendon excursion changes in response to equal applied force in various finger motion tasks in a normal pulley system. Our hypothesis was that the tendons would have different excursion changes (due to different contributions) in different tasks, including individual joint motions and free joint movement.

## Materials and Methods

### Specimen preparation

This study was approved and the need for consent was waived by the Institutional Review Board of National Cheng Kung University (NCKU) Hospital, TAIWAN. Ten fresh-frozen human cadaveric hands free from conspicuous musculoskeletal disease were amputated at the mid-forearm. Five males and five females with a mean age of 66.9 ± 10.3 (52–79) years were included. We used computer-generated random numbers to select five left hands and five right hands. The Asian Institute of TeleSurgery (AITS), Chang Bing Show Chwan Memorial Hospital (SCMH), TAIWAN, provided the cadavers. This study was part of a series of collaborative projects between NCKU and SCMH. The donated bodies were purchased from the LifeLegacy Foundation (http://www.lifelegacy.org) through the procurement system of Chang Bing SCMH, a branch hospital of SCMH. After thawing at room temperature, the specimens were dissected, and the FDS, FDP and extensor digitorum communis (EDC) of the long finger were identified. The Krakow technique with 1–0 Silk was used to make a suture at 5 cm proximal to the first crease of the wrist for tendon loading.

### Specimen mounting

**Fig 1:**
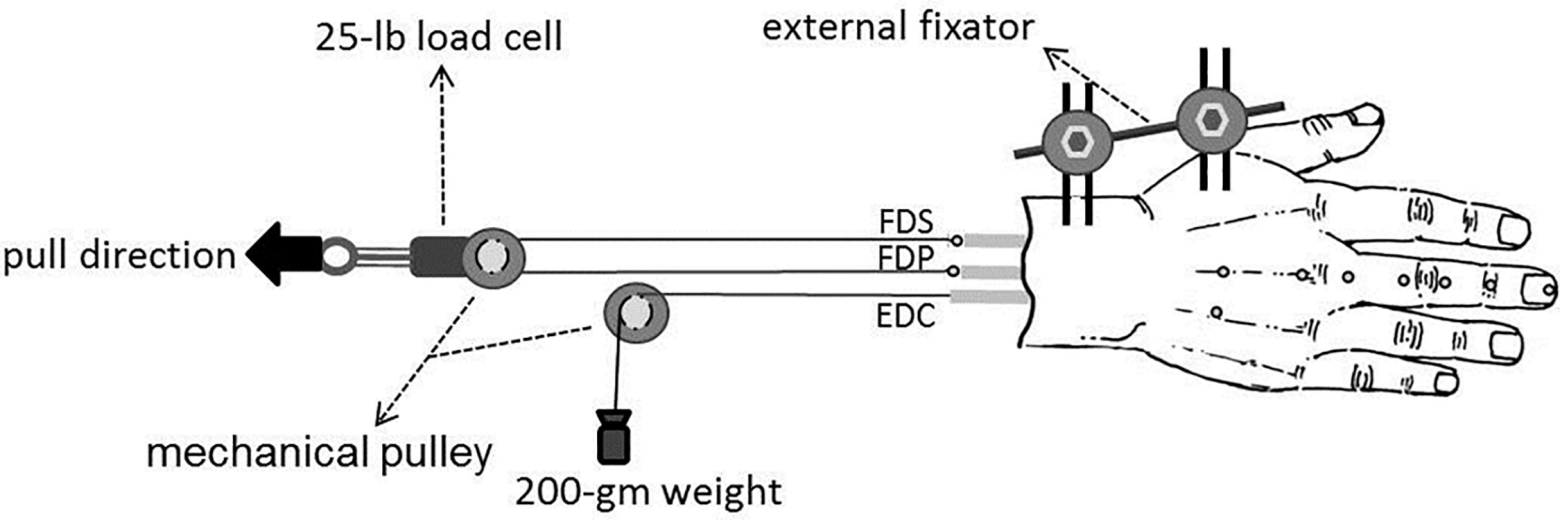
Illustration of the experimental setup with locations of the 2-mm–diameter reflective markers. The solid arrow indicates the direction in which the mechanical pulley was pulled. (FDS, flexor digitorum superficialis; FDP, flexor digitorum profundus; EDC, extensor digitorum communis)

Each wrist joint was fixed with an external fixator at the neutral position. Two parallel 2.5-mm–diameter Kirschner wires were inserted through the distal and proximal sections of the radius from the dorsal to volar aspects (four wires total) and then mounted on a custom-made fixation frame. After the targeted tendons were identified and sutured, the proximal ends of the FDS and FDP of the third finger were looped and linked with a Dacron line (1200 lb) through a mechanical pulley to build a synchronized system, which allowed the tendons to balance through different excursion changes as force was applied (Fig 1). Then, a 25-lb load cell (MDB-25, Transducer Techniques, Temecula, CA) was connected to the mechanical pulley for recording the acquired force. The mechanical pulley was attached to a two-dimensional slide-stand to ensure that the FDS and FDP moved simultaneously with the mechanical pulley without deviation. In addition, a 200-gm weight was secured by a Dacron line (1200 lb) at the end of the third EDC as a counterforce.

### Data collection

A synchronized motion analysis system and load cell captured the long finger’s motion trajectories and the acquired force respectively during motion. A three-dimensional Eagle Digit System (Motion Analysis Corporation, Santa Rosa, CA), which included eight Eagle Digital Cameras and the EagleHub and EVaRT software, recorded the trajectories at 100 Hz. Ten 2-mm–diameter retro-reflective markers (Fig 1) were attached to the examined hand’s dorsal aspect [4]. Two additional markers were placed on each tendon’s musculotendinous junctions (Fig 1) for tracking excursion changes.

Four joint motions (DIP, PIP and MCP joints individually and free joint movement) were tested. Finger splints at the neutral extended posture prevented other unnecessary joint movement to achieve individual DIP, PIP or MCP joint motion. However, no restrictions were applied during free joint movement. Before the mechanical pulley was pulled in each motion task, the weight in the EDC kept the finger at full extension. As the mechanical pulley was pulled manually to bring the finger to full flexion over a period of 10 seconds, the weight’s constant counterforce kept the motion balanced. Three trials were performed for each motion. A load cell connected to the mechanical pulley measured the acquired force, which was recorded simultaneously with each joint motion.

### Data analysis

Customizations of MATLAB (Version 7.14.0.739, The MathWorks, Inc., Natick, MA) handled all calculations. Kinematical and kinetic variables, including joint range of motion (ROM), tendon excursion and acquired loading force, were recorded and analyzed. The first fit moment arms (M) were tracked with Eq 1 for individual joint motion and generated from the slope of the linear region of the excursion-joint angle relationship scheme [9]. Each data point was an average of three trials, and all data were displayed as mean and standard deviation (SD). Additionally, all the force and joint angle change data were plotted in a motion trajectory and fit to a sigmoid regression function of force (Eq 2) using optimization to analyze the correlation of joint motion trajectories in free joint movement, where *x* is the normalized percentage of loading force, *x*_*o*_ is a location parameter related to the curve’s inflection point and *τ* is a slope parameter related to the angle change rate (the smaller the *τ* value, the steeper the slope).

$$S\left(x\right)=\frac{1}{1+e^{-\left(\frac{x-x_{0}}{\tau }\right)}}$$ (2)

Linear regression analyses determined the tendon excursions’ correlation in each motion. The regression line slopes indicate the tendons’ overall contributions throughout individual PIP motion, individual MCP motion and free joint movement. Moreover, at each instant of increased force, the FDP-to-FDS excursion ratio (P/S ratio) was calculated to determine each tendon’s contribution, and the excursions were measured in reference to the original point.

## Results

Loading force and changes in flexion angles and excursions in the full flexion of the long finger are recorded in Table 1. In individual joint motions, the loading force was transmitted by the mechanical pulley equally to each FDS and FDP to reach maximal flexion. The final force for the DIP, PIP and MCP individual unconstrained joint motions was 6.83 (SD 1.52) N, 8.79 (SD 1.69) N and 9.74 (SD 1.93) N respectively. The flexion angles of the starting positions of the DIP, PIP and MCP joints were at 10.91 (SD 4.89)°, 12.32 (SD 2.91)° and -12.13 (SD 9.41)° respectively; the maximal flexion positions were at 41.00 (SD 4.68)°, 91.92 (SD 12.42)° and 97.05 (SD 6.73)° respectively; and the ranges of motion (ROMs) were 30.09 (SD 7.89)°, 79.60 (SD 12.14)° and 109.18 (SD 14.32)° respectively. The FDP excursion changes were 10.13 (SD 1.79) mm, 21.91 (SD 3.29) mm and 29.75 (SD 4.15) mm for the DIP, PIP and MCP individual unconstrained joint motions respectively, and those of the FDS were 17.30 (SD 3.73) mm and 31.63 (SD 4.58) mm for the PIP and MCP individual unconstrained joint motions respectively.

**Table 1:** Loading force, flexion angles and excursion changes in full flexion of long finger. Values are mean (SD).

	Loading force to reach full flexion (N)	ROM (°)	Excursion (mm)	
			FDS	FDP
**Individual Joint Motions**				
**DIP**	6.83 (4.52)	30.09 (7.89)	N/A	10.13 (1.79)
		(10.91–41.00)		
**PIP**	8.79 (1.69)	79.60 (12.14)	17.30 (3.73)	21.91 (3.29)
		(12.32–91.92)		
**MCP**	9.74 (7.93)	109.18 (14.32)	31.63 (4.58)	29.75 (4.15)
		(-12.13–97.05)		
**Free Joint Movement**	8.15 (3.17)	109.18 (14.32)	39.35 (4.92)	43.56 (6.77)
		(-12.13–97.05)		

In free joint movement, the loading force transmitted by the mechanical pulley equally to each FDS and FDP to reach maximal flexion was 8.15 (SD 3.17) N. The sum of the joint angles at the starting position was 22.04 (SD 21.83)°, and the sum of the final maximal flexion angles was 231.99 (SD 15.83)°. The total ROM change was 209.96 (SD 33.99)°, and the FDS and FDP excursion changes were 39.35 (SD 4.92) mm and 43.56 (SD 6.77) mm respectively.

The FDS and FDP moment arms were calculated from linear regression fits (Table 2). The FDS and FDP linear moment arms across the MCP joint were 13.62 (SD 1.05) mm and 12.56 (SD 1.09) mm and across the PIP joint were 9.02 (SD 1.34) mm and 12.30 (SD 1.82) mm respectively. The FDP linear moment arm across the DIP joint was 10.43 (SD 0.23) mm.

**Table 2:** Moment arms of long finger calculated from linear fits. Values are mean (SD).

	ROM (°)	FDS		FDP	
		excursion (mm)	moment arm (mm)	excursion (mm)	moment arm (mm)
**DIP**	10.87 (0.11)	N/A	N/A	1.99 (0.16)	10.43 (0.23)
	(17.59–28.46)				
**PIP**	45.17 (0.56)	7.15 (1.22)	9.02 (1.34)	9.71 (1.59)	12.30 (1.82)
	(23.45–68.61)				
**MCP**	68.38 (0.55)	16.24 (1.35)	13.62 (1.05)	15.10 (1.11)	12.56 (1.09)
	(11.79–80.17)				

**Fig 2:**
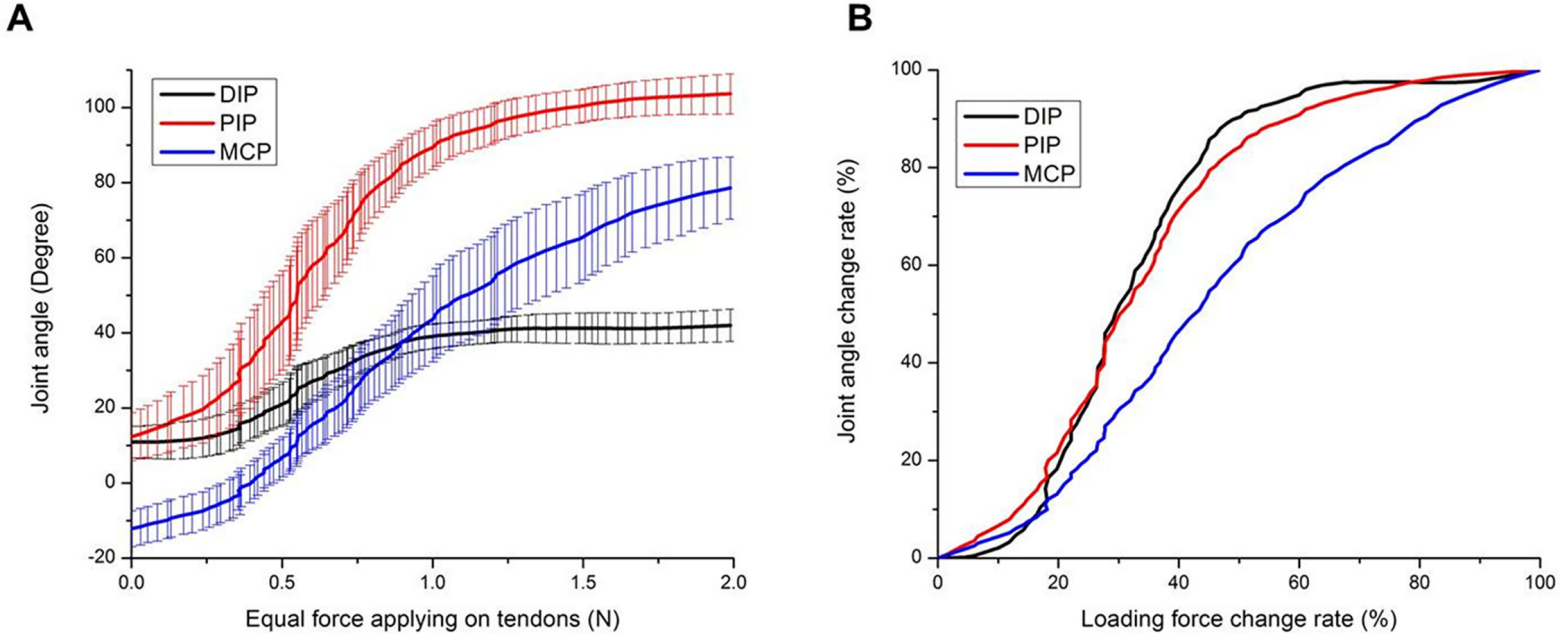
Group results of angular trajectories in full joint movement. (A) Correlation between absolute angle changes and applied force (bars represent standard deviation, SD). (B) Correlation between percentage of total angle change and percentage of total applied force.

To provide a basis for comparison of the specimens in free joint movement, the results had to be normalized on a percentage scale to account for individual specimen variations. The group results of the motion trajectories in free joint movement followed a gradually ascending S-curve (Fig 2A and 2B). The PIP and MCP joints initiated movement at low force. Then the PIP joint moved more quickly until 50% loading force, when it attenuated until maximal flexion. However, after initial movement, the MCP joint moved relatively constantly until maximal flexion. The correlation coefficient (*R*) of overall finger flexion between the PIP and MCP joints was 0.9388 (Fig 2B). DIP joint motion increased much more slowly than that of the PIP and MCP joints until 18% of the total loading force at a joint angle of 14.96 ± 5.27° for the DIP joint, 28.28 ± 11.09° for the PIP and -2.58 ± 5.20° for the MCP, at which point the motion of the DIP joint became relatively synchronized with that of the PIP. The *R* of overall finger flexion between the DIP and PIP joints was 0.9958 (Fig 2B). Moreover, the data of the parameters (*x*_*o*_ and τ) are shown in Table 3. The mean *x*_*o*_ of the MCP joint was significantly higher than those of the DIP and PIP joints (0.4 ± 0.13 vs. 0.3 ± 0.14, *p* = 0.025, and 0.32 ± 0.12, *p* = 0.040, respectively), and the mean τ of the MCP joint was significantly higher than those of the DIP and PIP joints (0.15 ± 0.05 vs. 0.07 ± 0.03, *p* < 0.001, and 0.10 ± 0.03, *p* = 0.009, respectively).

**Table 3:** Summary of the parameters (*x*_*o*_ and τ) of the sigmoid regression function for the optimization of the motion trajectory in free joint movement. Values are mean (SD).

Parameter	Joint	Mean (SD)	p-value
location parameter (*x*_*o*_)	DIP joint	0.30 (0.14)	0.025^a^
	PIP joint	0.32 (0.10)	0.040^b^
	MCP joint	0.44 (0.13)	
slope parameter (τ)	DIP joint	0.10 (0.03)	<0.001^a^
	PIP joint	0.07 (0.03)	0.009^b^
	MCP joint	0.15 (0.05)	

^a^pairwise comparison between the DIP and MCP joints

^b^pairwise comparison between the PIP and MCP joints

**Fig 3:**
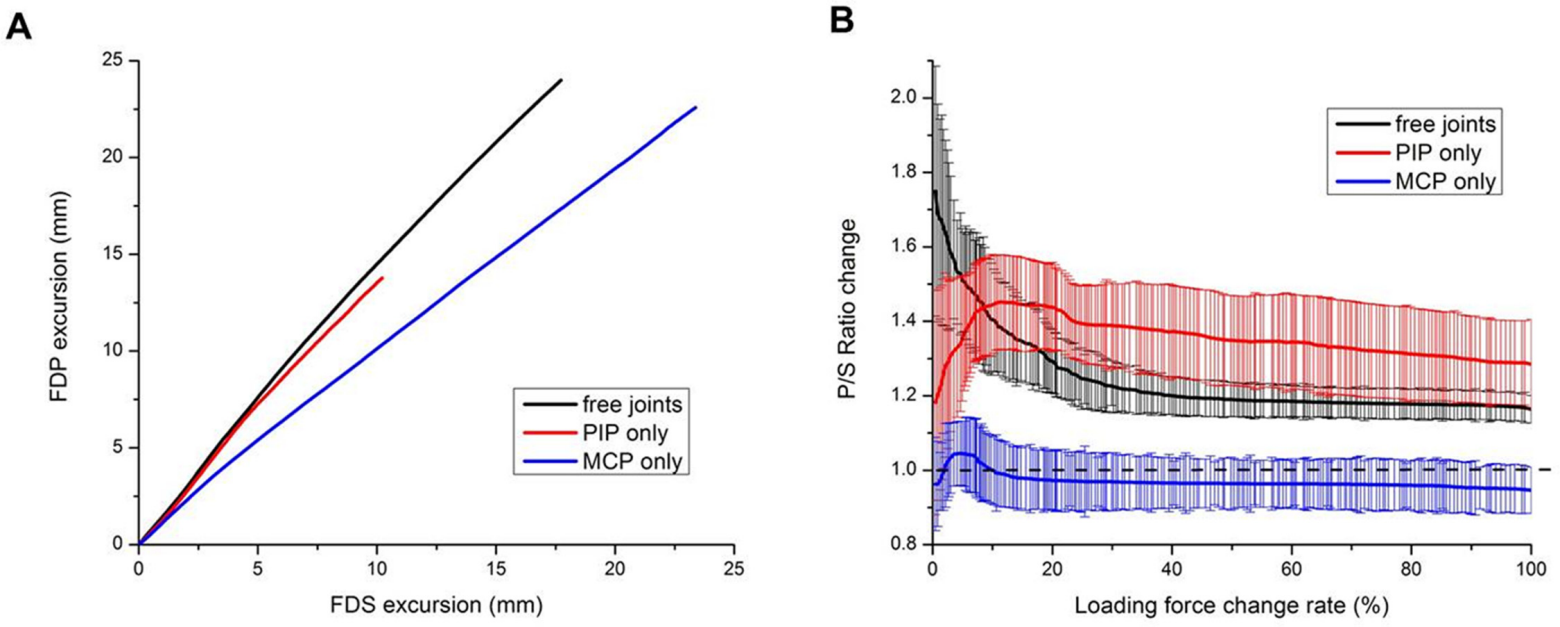
Group results of tendons’ contributions in different motion tasks. (A) Overall FDS-FDP contributions in individual PIP and MCP joint motions and free joint movement. (B) Correlation between the P/S ratio and applied instant force (expressed as a percentage of total force) in individual PIP joint motion, individual MCP joint motion and free joint movement.

To understand the contributions of each tendon’s excursion during joint motions, first, the linear regression slope of the group results was generated from the linear regression fitting with *R*-squared greater than 0.99 to discover their overall contributions. For individual unconstrained PIP and MCP joint motions alone, the FDP-over-FDS excursion slopes for the whole motion were 1.34 and 0.95 respectively (Fig 3A). In free joint movement, the slope was 1.37 (Fig 3A). Second, to understand the tendons’ contributions at each instant of increased force, the correlation between the P/S ratio and applied force (expressed as a percentage of total force) was analyzed. The results revealed that the FDP contributed more than the FDS (P/S ratio > 1.0) during the whole motion in individual PIP joint motion and free joint movement (Fig 3B). In individual PIP joint motion, the ratio reached a maximum of 1.45 at 11% force and 48.62 ± 13.31°. In free joint movement, the ratio started at the maximum and steadily declined until it reached a relatively constant ratio of about 1.2 after 50%. However, in individual MCP joint motion, the FDS contributed more (P/S ratio < 1.0) throughout the whole motion except between 1.9% and 13.1% force with a joint angle of -9.51 ± 6.59 ° and 22.04 ± 8.32 ° respectively (P/S ratio > 1.0), and the ratio reached a maximum of 1.05 at 7.2% force and 7.42 ± 7.07° and steadily declined until it reached a relatively constant ratio of about 0.96 after 43% (Fig 3B).

## Discussion

This study used a novel simultaneous system to exert equal force on the FDS and FDP using a looped mechanical pulley to reflect anatomical structures and physical conditions, i.e., the fact that the FDS and FDP both insert at the medial epicondyle of the humerus. Naturally, the FDS and FDP do not have exactly the same baseline characteristics, such as mass, volume and physiological cross-sectional area, and these factors are proportional to maximal tendon force and work capacity [22, 23]. However, regarding the relative contributions of the FDS and FDP to flexion and gripping, most studies have demonstrated that the two tendons have similar force ranges, averages and ratios. Range of force comparisons include 1.3 to 15.0 N and 3.1 N to 8.6 N for the FDS and 4.0 to 20.0 N and 4.0 N to 7.0 N for the FDP [24, 25]. Average tendon force comparisons include 10.4 N and 6.1 N for the FDS and 14.9 N and 5.7 N for the FDP [22, 26]. FDP-to-FDS force ratio results range from 0.7 to 1.1 [27, 28]. A few studies reported greater differences, such as an average tendon force of 0.60 N for the FDS and 4.00 N for the FDP and an FDP-to-FDS force ratio of 1.5 [22, 29]. The discrepancies between the various studies may relate to specimen differences, such as finger contours, finger postures and moment arms, and experimental design differences, such as counterweights, motion strategies and starting joint angles. Given that the majority of studies demonstrated a similarity between the tendons and that creating different ratios poses additional complexities in interpreting results, we decided that placing equal force through a mechanical pulley on each tendon was a suitable approach for this baseline study.

Using a looped mechanical pulley was necessary and more accurate than directly tying the tendons together because different muscles act on each tendon, pulling them in different proportions to achieve joint motion. This approach resembles an under-actuated robotic hand with pulley-based fingers. Such a hand uses two opposing fingers in an open-loop system to pinch and create a stable hold on an object [30–34]. The hand uses the length of the mechanical tendon as a basis for determining the most efficient arc for the finger and creates a closed kinetic chain movement by maintaining the minimum energy needed to create an adequate constraint relationship between the fingers and object [35–37]. Based on this concept, therefore, the mechanical pulley system in this study provides a zero-sum calculation that may replicate the inherent physiological efficiency and minimum energy consumption of tendon motion. Moreover, because the different muscles could not be used directly, equal force was applied to the mechanical pulley, and the tendons’ excursion changes could be adjusted to achieve the appropriate balance.

This study established the validity of our novel in *vitro* approach by comparing the moment arms and excursion changes with those of previous studies. The FDP’s loading force (8.15 ± 3.17 N) in free joint movement was within or less than the range of only single FDP tension (10–25 N) demonstrated in earlier studies [24, 29]. Moreover, the sum of full finger flexion ROM was 209.96 ± 33.99° (22.14 ± 16.24°, 92.42 ± 9.82° and 95.40 ± 16.31° for DIP, PIP and MCP respectively), less than the sum of 235.8° (73.64 ± 16.30°, 103.98 ± 8.98° and 85.30 ± 9.87° respectively) in Becker and Thakor [38] and more than the sum of 181° (63°, 75° and 43° respectively) in Kamper et al. [39]. But those studies’ motion data did not correspond to ours due to differences in the subjects and experimental designs.

The average linear moment arm across the PIP joint for the FDS and FDP respectively was 9.02 ± 1.34 mm and 12.30 ± 1.82 mm, and that across the MCP joint was 13.62 ± 1.05 mm and 12.56 ± 1.09 mm. In a comparison of these results to those of other studies, the relative error of the linear moment arm across the PIP joint for the FDS and FDP respectively was 3.66% and 3.98% (Franko et al.) and 0.22% and 17.7% (Armstrong and Chaffin). Additionally, the relative error of the linear moment arm across the MCP joint for the FDS and FDP respectively was 2.5% and 3.98% (Franko et al.) and 2.06% and 2.71% (Armstrong and Chaffin). Therefore, the results for the PIP and MCP joints are compatible with those of earlier studies [8, 40, 41]. However, the different insertion points of the FDS and FDP prevented head-to-head comparisons with these studies in individual unconstrained DIP joint motion because of the restriction of the PIP and MCP. Therefore, only the FDP worked on the DIP joint, decreasing the angle change and increasing the FDP’s moment arm (10.43 ± 0.23 mm). The moment arm, which is derived from tendon excursion and joint angle changes, is frequently tested in studies on finger motion. Therefore, it is a natural basis upon which to draw comparisons with established data. The compatibility of our results with existing data supported the basic reliability of this new approach.

The following can be concluded from the angular trajectories in free joint movement: (1) Less force was needed to actuate PIP and MCP flexion than DIP flexion. This makes sense considering that the FDS and FDP are two major extrinsic factors that concurrently flex the PIP and MCP joints with large passive torque, as opposed to the lower passive torque for the DIP, which only the FDP works on [8]. (2) DIP joint motion was delayed probably because the FDP’s moment arm was higher across the PIP joint than the DIP joint, although the FDP played a major role throughout. In addition, our finding that the FDS was more involved than the FDP early in flexion was consistent with those of other studies, such as the 2010 study by Li and Zhang, in which a computer model was used to predict the tendons’ contributions during flexion [12, 19, 42]. The results from our study and that of Li and Zhang were also consistent with those of the 1994 study by Greenwald et el., which showed that the FDP is not the primary flexor because, to initiate DIP movement, it has to overcome the EDC’s counterforce and rely on the pulley system to move a long distance efficiently [3, 12, 43, 44]. (3) After initial movement, the DIP continued more or less synchronously with the PIP joint, and these joints’ motions increased more quickly compared to the MCP’s steady increase. These findings are consistent with those of earlier studies [16, 17, 45] and can be explained by the need for the MCP’s torque to work against the interaction torques from the DIP and PIP joints since the inertial torque of the MCP arises from the motions of the DIP and PIP joints. Moreover, based on the results of the sigmoid regression, the motion trajectories of the DIP and PIP joints were similar, and these trajectories were significantly different from that of the MCP joint (i.e., the DIP and PIP joints had steeper slopes). Additionally, the correlation coefficient of the PIP and MCP joint motions was 0.9388. This finding is consistent with those of earlier studies [17, 19]. Finally, the DIP and PIP joints moved with a high correlation coefficient of 0.9958, in accordance with previous studies [16, 17, 45]. Therefore, in terms of the moment arms of the two tendons across different joints and the angular trajectories in flexion, this simultaneous approach was consistent with the physiological and kinematic characteristics demonstrated in previous studies that used different methodologies.

The second goal of our study was to provide information about the two tendons’ contributions unobtainable in previous approaches. Previous studies showed the simultaneous involvement of the FDS and FDP in finger flexion, especially in combined MP and PIP flexion at a medium or fast (200 ms or 400 ms) speed [16, 17] and in special tasks such as the downward and reciprocal finger motion phases of tapping [18]. However, these studies used electromyography (EMG), which cannot provide specific information about the contributions of the two tendons. In this study, first, we determined the linear regression slopes between the two tendons’ excursions in all motion tasks to understand the two tendons’ overall contributions. The FDP generally contributed more in individual PIP joint motion but less in individual MCP motion. These results reflect the tendons’ moment arms at these joints. Second, regarding the ratio at each instant of increased force, the FDP contributed more than the FDS during the entire individual PIP motion. However, in individual MCP motion, the FDP contributed less than the FDS except at 1.9–13.1% force, in accordance with the results of the EMG study by Darling et al. and Kuo et al. [17, 18]. In this case, the FDS plays a major role compared to the FDP. In free joint movement, the FDP contributed more than the FDS during the whole motion. These results reflect greater contributions of the DIP and PIP joint motions in flexion. In Darling et al. [16, 17], the FDP fired much more than the FDS early in flexion. However, FDS involvement increased afterward until the two tendons arrived at a relatively constant proportion. Thus, the FDS and FDP excursions were highly correlated during individual joint motions and free joint movement. Additionally, our results show that, despite the FDP’s dominance during the whole movement, the FDS played an increasingly greater supporting role as greater force was required [8, 12, 17, 39, 45]. Moreover, our study provides specific information for future research about the relationship between the FDS and FDP at different instants during flexion.

This study has some limitations. First, even though it has been demonstrated in an animal model that freezing does not affect the postmortem properties of soft tissue [46], differences between cadaveric and living tissue and between older and younger individuals may limit the breadth of the results’ application. Second, in real tasks, the muscles apply differing amounts of force rather than equal force to the tendons to maintain balance. However, applying equal force in this study was necessary because otherwise we could not have determined if the results reflected differing contributions of the tendons or the variable loading force. In the future, however, we plan to investigate different proportions of constant force applied to the tendons. Additionally, we did not monitor the level of force applied to individual tendons using a uniaxial force sensor [47]. In single joint posture movement, there is a linear relationship between the muscle force and the endpoint force [48, 49]. However, exertion that changes multiple joint postures has a nonlinear relationship with endpoint force, altering the tendon paths and joint motions and in turn the Jacobian matrix components and moment arms [49, 50]. In our study, we measured the exerted force only; therefore, in some instants, the endpoint force may not have been equally applied to the two tendons. In future studies, we plan to control for this difference. Third, the extensor mechanism and intrinsic muscles (e.g., the lumbricals and interosseous muscle) were not included and studied. This system may explain only certain tasks, such as power gripping, quick flexion and typing. Even in primarily sagittal motion, various muscles coordinate to produce the correct torque and position for each joint during motion tasks, and some joint motion tasks may not involve the same tendon mechanics. In biomechanical and EMG studies, various muscles, not only flexor tendons but also extensor and intrinsic muscles simultaneously involved in finger motion, are demonstrated. The extensor and intrinsic muscles may actuate early, but then the flexors take the primary role in flexion [51, 52]. The coordination of the extensor mechanism is a major consideration in biomechanical models because it could enhance the endpoint force magnitude. Most importantly, lumbricals and interosseous muscles are involved in certain motions by way of this extensor apparatus, causing the flexed MCP and extended interphalangeal joints to work together for grasping large objects and contributing to early MCP joint flexion [48, 51–54]. Despite the difficulty of obtaining useful data from the operation and analysis of complicated coordinated conditions, we plan to incorporate these coordinated conditions in future studies to increase the reliability of this approach. Fourth, although in *vitro* study allowed us to investigate certain aspects we could not in *vivo*, at this point, the difference between FDS-FDP contribution in *vitro* and in *vivo* is not yet clear. In the future, we plan to develop a further understanding of this issue through the ultrasonic speckled tracking technique in *vivo*.

## Conclusion

In conclusion, a novel in *vitro* approach was developed to simulate in *vivo* synchronized movements of the FDS and FDP. The findings include differences in finger performance and excursion amounts between the tendons in individual joint motions and free joint movement. This approach produced results mostly consistent with those of earlier studies. However, this approach also provided new information about the relative contributions of the tendons. We hope that such findings will provide important insights into future applications, for example, developing tendon gliding exercises for the treatment of mild and moderate cases of trigger finger and for rehabilitation after hand surgery, investigating the effects of different levels of pulley release on the flexor tendons’ contributions, and determining the flexor tendons’ contributions to the performance of other finger postures. As we continue to improve and build on this approach, our ultimate goal is to establish a research method that will facilitate hand disease observation and estimation, treatment plan development and evaluation in hand surgery and rehabilitation, and significant improvement of existing biomechanical hand models.
